# Plastic induced urinary tract disease and dysfunction: a scoping review

**DOI:** 10.1038/s41370-024-00709-3

**Published:** 2024-08-31

**Authors:** Liam O’Callaghan, Matthew Olsen, Lotti Tajouri, Davinia Beaver, Carly Hudson, Rashed Alghafri, Simon McKirdy, Adrian Goldsworthy

**Affiliations:** 1 Faculty of Health Sciences and Medicine, Bond University, Robina, QLD Australia; 2 Harry Butler Institute, Murdoch University, Murdoch, WA Australia; 3 Dubai Police Scientists Council, Dubai Police, Dubai, United Arab Emirates; 4 International Centre for Forensic Sciences, Dubai Police, Dubai, United Arab Emirates

**Keywords:** Microplastic, Nanoplastic, Kidney, Urine, Urinary tract, Incontinence, Inflammation

## Abstract

**Introduction:**

In 2019 the World Health Organisation published a report which concluded microplastics in drinking water did not present a threat to human health. Since this time a plethora of research has emerged demonstrating the presence of plastic in various organ systems and their deleterious pathophysiological effects.

**Methods:**

A scoping review was undertaken in line with recommendations from the Johanna Briggs Institute. Five databases (PubMed, SCOPUS, CINAHL, Web of Science and EMBASE) were systematically searched in addition to a further grey literature search.

**Results:**

Eighteen articles were identified, six of which investigated and characterised the presence of microplastics and nanoplastics (MNPs) in the human urinary tract. Microplastics were found to be present in kidney, urine and bladder cancer samples. Twelve articles investigated the effect of MNPs on human cell lines associated with the human urinary tract. These articles suggest MNPs have a cytotoxic effect, increase inflammation, decrease cell viability and alter mitogen-activated protein kinases (MAPK) signalling pathways.

**Conclusion:**

Given the reported presence MNPs in human tissues and organs, these plastics may have potential health implications in bladder disease and dysfunction. As a result, institutions such as the World Health Organisation need to urgently re-evaluate their position on the threat of microplastics to public health.

**Impact statement:**

This scoping review highlights the rapidly emerging threat of microplastic contamination within the human urinary tract, challenging the World Health Organisation’s assertion that microplastics pose no risk to public health. The documented cytotoxic effects of microplastics, alongside their ability to induce inflammation, reduce cell viability and disrupt signalling pathways, raise significant public health concerns relating to bladder cancer, chronic kidney disease, chronic urinary tract infections and incontinence. As a result, this study emphasises the pressing need for further research and policy development to address the challenges surrounding microplastic contamination.

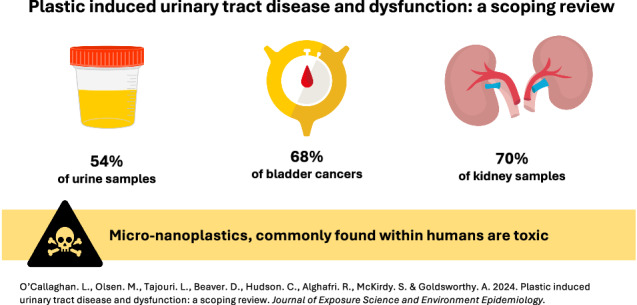

## Introduction

Since their widespread adoption in the mid-20th century, plastics have transformed from a novel substance into a versatile range of materials that pervade all aspects of society. The extensive integration of plastics into all industries and facets of society led to the production of 368 million tonnes of plastic in 2019, a figure expected to double by 2039 [1, 2]. The production and degradation of plastic, a process involving a combination of physical, chemical and biological processes, has resulted in the accumulation of miniscule fragments termed microplastics (MPs) or nanoplastics (NPs) [3], residing within all aspects of our environment including flora [4] and fauna [5–7], streams and oceans [8], air [9], and soil [10]. Beyond the well-documented environmental impacts of MPs and NPs, evidence for their presence within human organ systems and their role in driving various pathophysiological processes and diseases is rapidly emerging [11–15]. Of notable concern is their presence within the urinary tract and consequent impact on renal and bladder disease and dysfunction, as seen in animal studies [16, 17].

As the impact of plastic pollution becomes increasingly evident, so too does the need for internationally standardised definitions of MPs and NPs. Government publications reveal notable discrepancies in the definition of such fragmented plastics between organisations. For example, MPs are described by the European Union as “…small pieces of plastics, usually smaller than 5 millimetre (mm)”, and there is no formal definition provided for NPs [18]. The National Science Foundation describes MPs and NPs as fragments less than 5 mm and less than 100 nanometre (nm), respectively [19]. In contrast, the International Organisation for Standardisation (ISO) offers a more detailed classification, defining MPs as solid plastic particles insoluble in water, ranging from 1 micrometre (µm) to 1000 µm (1 mm), and NPs as particles smaller than 1 µm [20]. This variation and lack of consensus across different regulatory bodies exemplifies the challenges associated with mitigating the environmental and health-related impacts of microplastics and nanoplastics (MNPs).

Following their intrusion into the human body, whether through inhalation, ingestion, or through the skin (wound, hair follicle, sweat gland) [21–23], MNPs have been observed to cross biological barriers, leading to systemic exposure, and resulting in their detection in multiple critical organ systems [24]. To date, the presence of MNPs has been demonstrated to have deleterious pathophysiological implications resulting in inflammation, alterations in cellular metabolism, mechanically induced cellular damage and decreased cell viability [25–27].

Pathologies of the urinary tract, partially resulting from issues surrounding increasing antimicrobial resistance, rates of cancer and ageing populations, result in significant health-related economic expenditure, morbidity and mortality. Globally, an estimated 404.61 million urinary tract infections (UTIs) occurred in 2019 alone, resulting in over 236 000 deaths and 520 000 disability adjusted life years (DALYs) [28]. The cost of renal replacement, while only utilised by 0.15% of the population, represents 2–4% of total healthcare expenditure in some countries [29]. As a result, the detection of MNPs in the human urinary tract, in conjunction with a lack of understanding regarding their effects, currently provides reason for concern within the healthcare community. These concerns are potentially augmented by foreseeable difficulties in the removal of plastics from organs and the treatment of plastic-associated diseases.

Therefore, this scoping review aims to systematically explore and summarise the literature regarding the presence of MNPs in the human urinary tract and their pathological consequences, and explore the methodologies used in their detection and analysis, guided by the following research questions:

**RQ1:** What are the characteristics of plastics which have been found in the human urinary tract?

**RQ2:** How are MPs and NPs currently defined within the literature?

**RQ3:** What methodologies are currently utilised to explore the presence of MNPs and their effects?

**RQ4:** What are the pathophysiological consequences of the presence of MNPs in the human urinary tract?

For the purpose of this review, a broad definition of the term ‘urinary tract’ will be employed, inclusive of the kidneys, bladder, ureter, urethra, and urine. This will ensure that a broad scope of the literature is undertaken which may assist in elucidating the complex interactions between plastic in urine and adjacent structures. By focusing on the urinary tract, this review attempts to solidify the field’s understanding of MNPs, raise awareness of this important emerging issue, and lay the foundation for further research which may assist in the development of public health policies and clinical practice guidelines.

## Methods

### Protocol and registration

An a priori protocol (https://osf.io/wjk9u) was developed in line with the recommendation of the Joanna Briggs Institute (JBI) and the PRISMA extension for scoping reviews reporting guidelines (PRISMA-ScR). This protocol was published on the Open Science Framework on the 20/02/2024.

### Eligibility criteria

A pre-determined eligibility criteria was developed, informed by the population (human), concept (microplastics or nanoplastics and their effects) and context (urinary tract). Pre-determined definitions were developed and outlined within the a priori protocol after careful evaluation of the existing literature. For clarity, plastics were defined as a synthetic or semi-synthetic material comprising organic polymers from plant extracts or fossil fuels. The term ‘urinary tract’ was defined, in line with the National Institute of Diabetes and Digestive and Kidney Diseases, as a system of organs, including the kidneys, ureter, bladder, and urethra, responsible for the production, storage and excretion of urine [30]. To ensure a broad and thorough scope of the literature was undertaken, all research methodologies and abstracts were included with the exception of narrative reviews. Pre-print articles were included following a critical review of the article’s methodology by authors experienced in laboratory methodologies.

### Search strategy

A search strategy was developed utilising a three-step methodological approach originally proposed by Arksey and O’Malley [31] and further outlined by the JBI [32]. Firstly, a pilot search of PubMed and Google Scholar was undertaken on the 19/01/2024. Secondly, results were reviewed to identify additional search terms, with the final search strategy being translated for additional search engines with the assistance of a validated search engine translation software (Systematic Review Accelerator [SRA] Polyglot [33]) (Appendix 1). An additional search for grey literature was undertaken utilising Research Rabbit [34], TERA Farmer [35] and Perplexity [36].

### Information sources

Five databases (PubMed, EMBASE, CINAHL, SCOPUS and Web of Science) were searched on the 15/02/2024. Results from database searches were exported in EndNote X9 [37].

### Selection of sources of evidence

Duplicate results were removed utilising automation software (SRA Deduplicator) [38]. Articles were screened by two authors (AG and LO) by title and abstract within SRA Screenatron [38]. Full text screening was undertaken within Covidence [39] by two authors (AG and LO). Due to a lack of disagreement, a third author was not required to resolve disputes.

### Data charting of data items

A draft extraction table was developed within Microsoft Excel to align with the aims of the scoping review, which was piloted and refined prior to two authors (AG and LO) undertaking full data extraction. Where information was not relevant or not reported, this was recorded for clarity.

### Synthesis of results

Data pertaining to definitions was extracted, and where possible, synthesised to assist in analysis. Data pertaining to the countries and years of publication was tabulated and visually represented. To assist in evaluation, studies identifying the presence of plastic in human specimens, and studies evaluating the effects of plastic on cellular viability, uptake, and function have been tabulated separately.

## Results

### Selection of sources of evidence

**Fig. 1:**
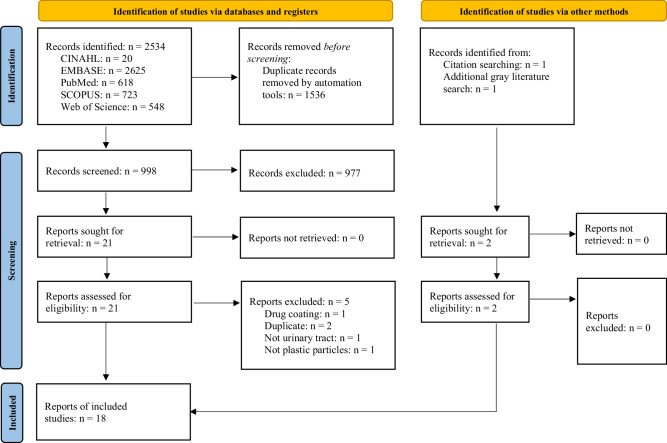
PRISMA-ScR Flow Diagram.

Database searching led to the retrieval of 2534 articles, of which 1536 articles were removed via automation. Title and abstract screening of the remaining 998 articles led to the exclusion of a further 977 articles. The full text of the remaining 21 articles was successfully retrieved and screened with substantial agreement between authors (Cohen’s Kappa = 0.765), leading to the exclusion of a further five articles [40]. A final grey literature search revealed two additional articles, one of which was a pre-print (Fig. 1).

### Synthesis of results

**Fig. 2:**
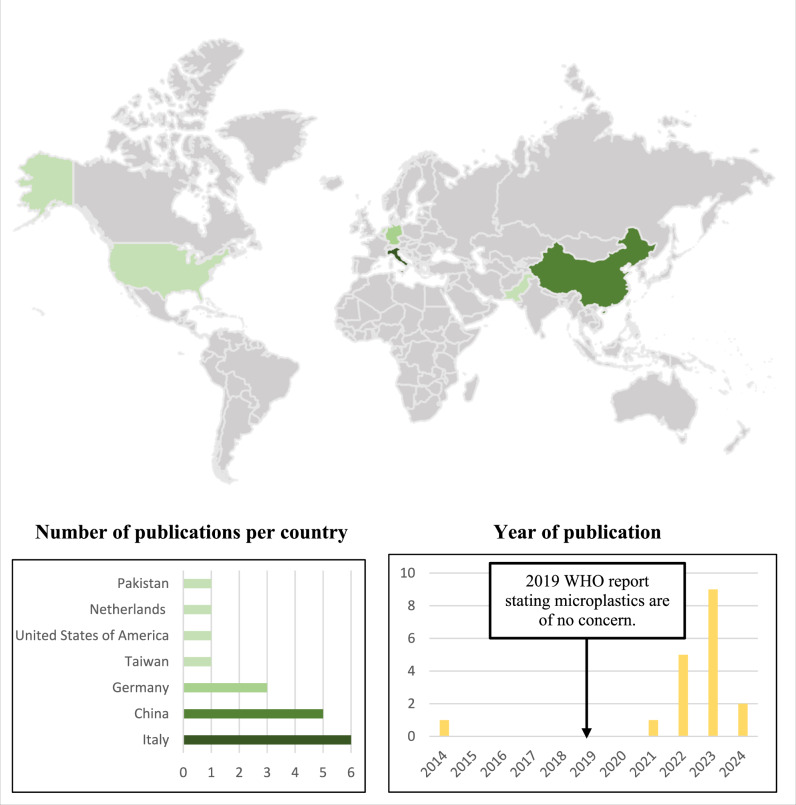
Article characteristics. Details pertaining to country of origin, number of publications per country and year of publication.

**Fig. 3:**
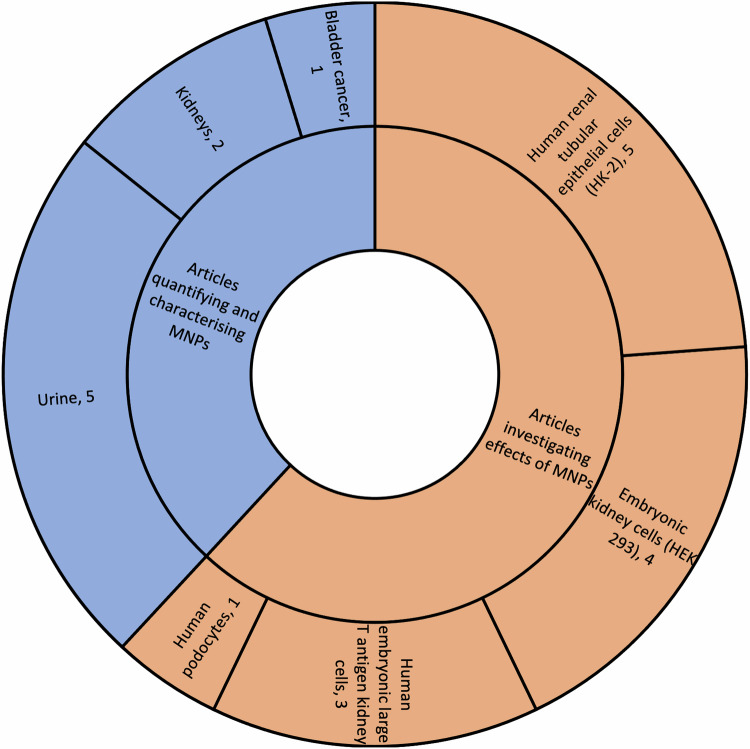
Number of articles organised by aim of article and sample type.

Of the 18 identified articles, the authorship groups represent seven countries: Pakistan (*n* = 1), Netherlands (*n* = 1), the United States of America (*n* = 1), Taiwan (*n* = 1), Germany (*n* = 3), China (*n* = 5), and Italy (*n* = 6). Only one identified article was published prior to 2022 (Fig. 2). Six articles (Fig. 3) identified and characterised the presence of MNPs in human urine (*n* = 5), human kidney samples (*n* = 2), and bladder cancer (*n* = 1). In addition, 12 articles investigated the effect of MNPs on human renal tubular epithelial cells (HK-2) (*n* = 5), human embryonic kidney cells (HEK 293) (*n* = 4), human embryonic kidney SV40 large T antigen cells (293T) (*n* = 3) and human podocytes (*n* = 1).

### Definitions of microplastics and nanoplastics

Only 12 out of the 18 articles provided a description of MPs or NPs (Table 1). The size of MPs varied between 0.1 μm and 5 mm. In addition, six out of the 12 articles differentiated between MPs and NPs. Descriptions of NPs varied between 1 μm and <0.1 μm.

**Table 1:** Details pertaining to definitions of microplastics and nanoplastics listed in order of publication.

Author	Country	Month and year of publication	Definition
Chen et al. [44]	Taiwan	April 2022	*Microplastics* are particles, debris, or fragments smaller than 5 mm that originate from the degradation of larger plastic items or from direct sources like microbeads in cosmetics.
Zhang et al. [47]	China	May 2022	*Microplastics* plastic particles < 5 mm and *nanoplastics* < 1 μm.
Goodman et al. [52]	United States of America	September 2022	*Microplastics* are less than 5 mm in size.
Xiao et al. [46]	China	January 2023	*Microplastics* are plastics with a size of 0.1–5000 μm and can degrade to the nanoscale, which is called *nanoplastics*.
La Porta et al. [42]	Italy	March 2023	*Microplastics* are fragments less than 5 mm in diameter.
Cervello et al. [50]	Italy	June 2023	*Microplastics* are plastic fragments between 1 μm and 5 mm in diameter with *nanoplastics* being <1 μm.
Exacoustos et al. [54]	Italy	June 2023	*Microplastics* are plastic fragments <5 mm in diameter.
Li et al. [45]	China	July 2023	*Microplastics* < 5 mm and *nanoplastics* < 1 μm.
Barnett et al. [99]^a^	Pakistan	July 2023	*Microplastics* encompass a range of small fragments, fibres, films, and granules composed of synthetic polymers or polymer matrices. They typically have a diameter of 1 μm up to 5 mm and can originate from both primary and secondary sources.
Zhu et al. [43]	China	November 2023	*Microplastics* < 5 μm and *nanoplastics* < 0.1 μm.
Massardo et al. [55]	Italy	February 2024	*Microplastics* are plastic particles with sizes between 1 μm and 5 mm.
Song et al. [41]	China	March 2024	*Microplastics* are 0.1 μm to 5 mm and *nanoplastics* are <0.1 μm.

^a^Preprint.

### Identification techniques and characteristics of microplastics and nanoplastics

**Fig. 4:**
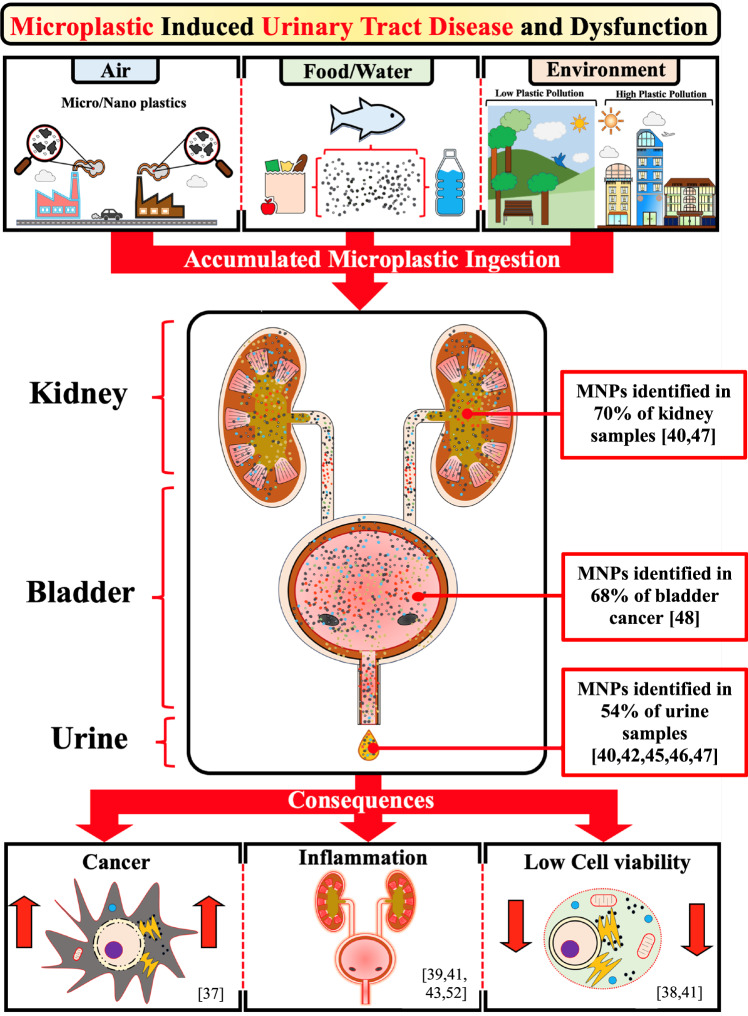
Microplastic bioaccumulation. Summary of consequences of microplastic bioaccumulation pertaining to the urinary tract.

**Fig. 5:**
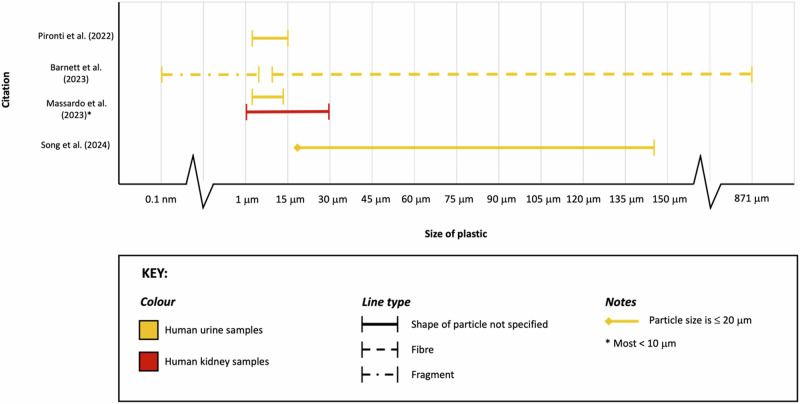
Size comparison. Size of plastic organised by sample type and particle shape.

The most commonly utilised method for detecting and characterising MNPs in human samples was Raman spectroscopy (5/6; 83%) (Fig. 4). Alternatively, Song et al. [41] utilised pyrolysis-gas chromatography mass spectroscopy combined with laser direct infrared spectroscopy. Four articles described the number of samples in which MNPs were identified (Table 2). MNPs were identified in 15/28 (54%) of the human urine samples, 13/19 (68%) of the human bladder cancer samples and 7/10 (70%) of the human kidney samples. Both MNP fibres and particles were identified within specimens with large variations in size, from 0.1 nm to 871 μm (Fig. 5). Thirteen types of polymers were identified within the samples. Polystyrene was the only polymer which was identified across urine, kidney and bladder cancer samples (Table 2).

**Table 2:** Details of articles investigating the presence of MNPs in samples from humans.

Author (Year) Country	Sample type	Analysis approach	Characteristics of Plastic				
			Quantity	Shape	Size	Colour	Polymer Matrix
Pironti et al. (2022) Italy [100]	Human urine	Raman spectroscopy	8 fragments identified from 4/6 samples	7 irregular 1 spherical	~4 μm to ~15 μm	Transparent Brown Blue Blue/grey Green Red	Polyethylene vinyl acetate Polyvinyl chloride Polypropylene Polyethylene
Massardo et al. (2023) Italy [101]	Human urine Kidney	Raman spectroscopy	Urine samples: 23 Kidney samples: 43	Not described	Urine: 3 μm–13 μm Kidney: 1 μm–29 μm Note: most samples <10 μm	Light blue Red Orange Colourless Dark blue Brown Black Grey	Polyethylene Polystyrene Styrene-isoprene
Krafft et al. (2022) Germany [102]	Bladder cancer resectates	Raman spectroscopy	13/19 specimens contained microplastics	Not described	Not described	Not described	Polyphenylene sulphide Polystyrene
Barnett et al. (2023) Pakistan [99]	Human urine	Raman spectroscopy Scanning electron microscopy	Microfibers: 2.04 ± 3.38/100 mL Fragments: 1.59 ± 1.80/100 mL	Fibres and particles	Fibre length: 10–871 μm Fragment size: 0.01 nm–6 μm	Not described	Polyamide Polypropylene
Song et al. (2024) China [41]	Human urine	Pyrolysis-gas chromatography/mass spectrometry Laser direct infrared spectroscopy	15 plastic particles identified from 7/12 samples.	Fibres and spherical particles	≤20 μm–144.04 μm	Not described	Acrylates (33.67%) Polymethylmethacrylate (23.92%) Polyurethane (17.13%) Polypropylene (15.92%) Polyethylene terephthalate (9.36%)
Exacoustos et al. (2023) Italy [54]	Human urine Kidney	Raman spectroscopy	Human kidney: 17 fragments identified from 7/10 samples. Human urine: 9 fragments identified from 7/10 samples.	Not described	Not described	Not described	Polystyrene Styrene-isoprene Polyethylene

### In vitro exposure of MNPs in renal cells

A significant proportion of studies (9/12 or 75%) used fluorescence labelling to track the internalisation of MNPs in renal cells, suggesting that fluorescence-based techniques are commonly employed for the visualisation and quantification of MNP uptake (Table 3). The primary techniques across studies varied, but included cell viability assays, microscopy, and flow cytometry. Interestingly, almost all studies (8/12 or 67%) used polystyrene as the model for MNPs, with size ranging from 20 nm to 3.39 μm. Additionally, 3/12 of the studies (27%) utilised polyethylene, and one study used a combination of polyvinyl chloride (PVC), polypropylene (PP), polyamide (PA), and tyre wear particles. Moreover, 8/12 of the studies (67%) described the shape of the MNPs used as spherical. Most studies used human renal tubular epithelial cells (HK-2) (5/12 or 42%) and human embryonic kidney cells (HEK 293 and HEK 293T) (6/12 or 50%), with one study using human podocyte cells. Common endpoints included cell viability, inflammatory response, oxidative stress, apoptosis, and changes in MAPK and PI3K-AKT signalling pathways. The exposure of MNPs to renal cells resulted in decreased cell viability [42–45], increased inflammation and activation of potentially cancer-inducing pathways [43, 46]. One study demonstrated that MNPs facilitated the infection of HEK 293T cells with SARS-CoV-2 [47].

**Table 3:** Details of articles investigating the impact of MNPs on human cell lines.

Author (Year) Country	Research aim/question	Sample details	Analysis	Key findings
Richards et al. (2023) Netherlands [83]	*RQ*: How many nanoparticles enter cells and how rapidly do they do so?	*Cell Line*: - HEK 293 *Plastic Model*: - Fluorescent-labelled carboxylated polystyrene (40–200 nm) - Shape: Spheres	- Live-cell confocal microscopy - Super-resolution Stimulated Emission Depletion microscopy - Flow cytometry	- Majority of NPs (40–200 nm) desorb from HEK 293 cell membranes within 1–2 s after adsorption. - Most particles that are internalised enter the cell within 1–2 s, independently of particle size. - Rapid internalisation is suggested to occur via an endocytic event already taking place or through an uncharacterised endocytic route.
Xiao et al. (2023) China [46]	*Aim*: Assess toxicity of polystyrene NPs in kidney and testis via a kidney-testis microfluidic platform.	*Cell Line*: - HK-2 *Plastic Model*: - Fluorescent-labelled polystyrene (50 nm) - Shape: Spheres	- Cell viability - Glucose consumption - Transmission electron microscopy - Immunofluorescence staining - ELISA	- Polystyrene NPs were internalised by HK-2 cells via endocytosis. - Activation of cancer-related signalling pathways was triggered by polystyrene NPs (MAPK & PI3K-AKT).
Zhu et al. (2023) China [43]	*Aim*: Investigate the renal toxicity of polystyrene NPs and the underlying mechanisms of their effect on human health.	*Cell Line*: - HK-2 *Plastic Model*: - Polystyrene (20, 50 nm) - Shape: Not described	- Cell viability - Lactate dehydrogenase - Transmission electron microscopy - Flow cytometry - Measurement of ROS - Mitochondrial membrane potential assessment - Endoplasmic reticulum staining - Transcriptional and RT-PCR - Western blot	- Polystyrene NPs exposure induced apoptosis in HK-2 cells in a dose-dependent manner, with 20 nm polystyrene NPs at 100 μg/mL causing both early and late apoptosis. - NPs increase ROS and alter mitochondria and Endoplasmic reticulum structures. - MAPK signalling pathway activated, with increased expression levels of phosphorylated p38 and ERK1/2, and induced inflammation and apoptosis in HK-2 cells.
Yarbakht et al. (2021) Germany [51]	*Aim*: Evaluate the effects of microplastics including polycinylchloride, polpropylene, polyamide and tyre wear particles on the viability and morphology of human podocytes in vitro.	*Cell Line*: - Human podocytes *Plastic Model*: - Polyvinyl chloride, polypropylene, polyamide & tyre wear particles - Shape: Not described	- Cell viability - Phalloidin staining - Raman spectroscopy - Scanning electron microscopy	- Cytotoxicity of particles on podocytes depends on polymer type. - Particle attachment varies with adhesion properties. - Particles induce cytoskeleton reorganisation. - Exposure duration and particle concentrations are key factors in evaluating the toxicological effects of particles on podocytes, with particle attachment on the cell surface and uptake of smaller particles. through phagocytosis being potential mechanisms of harm.
Firdessa et al. (2014) Germany [84]	*Aim*: Investigate whether a single cell type employs several uptake mechanisms simultaneously to internalise a given type of NPs.	*Cell Line*: - HEK 293T *Plastic Model*: - Polystyrene latex nanoparticles (20, 100, 200, 500 nm). - Shape: Spheres	- Cell viability - Flow cytometry - Macrophage infection by Leishmania major - Transmission electron microscopy - Fluorescence and confocal microscopy	- NPs are rapidly internalised and accumulate in endosomal compartments. - The internalisation process is energy-dependent. - Uptake depends on NPs size, cell type, and exposure time. - Multiple endocytosis pathways facilitate the uptake of NPs.
Li et al. (2023) China [45]	*Aim*: Explore toxicity of polystyrene NPs to HEK 293T cells with a focus on the effects of particle sizes and Pb2+ enrichment.	*Cell Line*: - HEK 293 T *Plastic Model*: - Pristine polystyrene & fluorescent-labelled polystyrene (20, 60, 100, 500, 1000 nm) - Shape: Spheres	- Cell viability - Confocal microscopy - Measurement of ROS - Measurement of ATP - Lipid peroxidation - Measurement of lactate dehydrogenase - qRT-PCR	- NPs toxicity is size- and concentration-dependent. - 20 nm NPs reduced viability to 53.0%; with Pb2+, dropped to 26.7%. - 60 nm NPs reduced viability to 74.8%; with Pb2+, dropped to 50.8%. - Pb2+ on NPs increases toxicity. - NPs induce oxidative damage and inflammation in cells. - Exposure to NPs and Pb2+ triggers cell apoptosis
Chen et al. Taiwan [44]	*Aim*: Determine the nephrotoxic potential of polystyrene MPs at realistic environmental concentrations.	*Cell Line*: - HEK 293 *Plastic Model*: - Polystyrene (~3.39 ± 0.30 μm). - Shape: Sphere	- Cell viability - Phase-contrast microscopy - DCFH-DA - JC-1 probe and DAPGreen staining - Quantibody® Human Inflammation Array 3 Kit - Western blot	- Polystyrene MPs adhered to and internalised in HEK 293 cells. - Exposure to polystyrene MPs at 30 and 300 ng/mL induced cytotoxicity and morphological changes. - Triggered autophagy and apoptosis in HEK 293 cells. - Nephrotoxicity was observed at both noncytotoxic and cytotoxic concentrations.
Beltrame et al. Italy [49]	*Aim*: Evaluate the effects of Bisphenol A and polyethylene microplastics, as well as their combination, on HK-2 cells.	*Cell Line*: - HK-2 *Plastic Model*: - Polyethylene (1–4 μm) - Shape: Not described	- Microscopy - MTT - RT-PCR - Western blot - Immunofluorescence	- Exposure to polyethylene MPs and bisphenol A reduces cell viability. - Increases expression of inflammatory molecules. - Induces a pro-inflammatory response in renal tubular cells. - Combined effect of bisphenol A and MPs is worse than either alone.
Cervello et al. Italy [50]	*Aim*: Evaluate the toxicity of polyethylene and bisphenol-A MPs on HK-2 cells in vitro.	*Cell Line*: - HK-2 *Plastic Model*: - Polyethylene - Shape: Sphere	- Proteomic analysis by mass spectrometry	- Proteomic analysis differentiated the HK-2 proteome based on conditioning. - Identified a “core” of significant proteins: Nephronectin, GDF15, Vasorin, IGFBP7, Midkine, and Tissue factor-F3. - Markers of stress conditions, including inflammation and oxidative stress.
Goodman et al. (2022) United States of America [52]	*Aim*: Investigate the adverse effects that MPs have on cellular morphology, proliferation, stress, metabolism and internalisation in HEK 293 cells.	*Cell Line*: - HEK 293 *Plastic Model*: - Fluorescent-labelled polystyrene (1 μm) - Shape: Sphere	- Cell viability - Phase-contrast microscopy - MTT - Cell proliferation - Confocal fluorescence microscopy - Flow cytometry - Measurement of ROS - EdU cell proliferation assay - qRT-PCR	- 1 μm polystyrene MPs reduced cell proliferation. - Caused morphological changes in HEK 293 cells. - Increased reactive oxygen species (ROS) levels in exposed cells. - Lowered gene expression of key enzymes.
La Porta. (2023) Italy [42]	*Aim*: Evaluate the in vitro toxicity of polyethylene MPs on HK-2 cells.	*Cell Line*: - HK-2 *Plastic Model*: - Pristine polyethylene, fluorescent-labelled polyethylene (1–4 μm) - Shape: Sphere	- Raman spectroscopy - MTT - Immunocytochemistry - Western blot - mRNA spectrophotometry - Proteomic analysis by mass spectrometry	- Exposure to MPs and bisphenol-A reduced cell viability. - Significant upregulation of MCP-1, IL-1b, AhR, and NOX-4. - Downregulation of HSP90. - NRF2 was downregulated by bisphenol-A and upregulated by MPs.
Zhang et al. (2022) [47]	*Aim*: Examine the interaction of MPs and SARS-CoV-2 including the uptake of virus coated MP by cells.	*Cell Line*: - HEK 293T *Plastic Model*: - Polystyrene - Shape: Sphere	- Fluorescence microscopy - Confocal microscopy - Flow cytometry	- MP adsorb SARS-CoV-2 on their surface by shuttling the virus into the endo-lysosomal compartment.

*NPs* nanoplastics, *HEK 293* human embryonic kidney 293 cells, *HEK 293T* human embryonic kidney SV40 large T antigen cells, *HK-2* human renal tubular epithelial cells, *ELISA* enzyme-linked immunosorbent assay, *MAPK* mitogen-activated protein kinase, *PI3K-AKT* phosphoinositide 3-kinase/protein kinase B, *ROS* reactive oxygen species, *PCR*, polymerase chain reaction, *MPs*, microplastics, *DCFH-DA* 2’,7’-dichlorodihydrofluorescein diacetate, *JC-1* 5,5’,6,6’-Tetrachloro-1,1’,3,3’-tetraethylbenzimidazolylcarbocyanine iodide, *MTT* (3-(4,5-Dimethylthiazol-2-yl)-2,5-diphenyltetrazolium bromide), *RT-PCR* reverse transcription polymerase chain reaction, *ATP* adenosine triphosphate, *MCP-1* monocyte chemoattractant protein-1, *IL-1b* interleukin 1 beta, *AhR* aryl hydrocarbon receptor, *NOX-4* NADPH oxidase 4, *HSP90* heat shock protein 90, *NRF2* nuclear factor erythroid 2-related factor 2.

## Discussion

To the best of our knowledge, this scoping review provides the first effort to collate and synthesise research investigating the presence and effect of MNPs on the human urinary tract. Considering the World Health Organisation’s (WHO) 2019 statement entitled ‘Microplastics in drinking water’, which downplays the significance of MPs in drinking water, the findings of articles included within this scoping review challenge this position [2]. The WHO’s assertion, made in a 124-page report, that “there is no evidence to indicate a human health concern” is increasingly contradicted by emerging research [2]. Since this report, and a call for further research to be conducted by the WHO, there has been a 15-fold increase in the number of publications made investigating the harmful impacts of MNPs [48] with numerous publications demonstrating harmful effects [43, 45, 49, 50]. The articles identified in this scoping review reported MNPs led to increased inflammation [43, 45, 49, 50], cellular toxicity [42–45, 49, 51], and disruption to normal physiological processes [43, 46, 52]. While not the focus of this scoping review, an even greater number of animal studies report similar findings highlighting MNP exposure as a threat to One Health [53]. This challenges the WHO’s initial assessment and highlights the urgent need for revaluation of MNPs impact on human health.

### Defining micro-nano-plastics

Significant inconsistencies exist between definitions of MPs and NPs within academic literature, in addition to those proposed by industrial bodies and government authorities. The majority of articles [41, 42, 44, 45, 50, 52, 54, 55] adopt a size-related definition of MPs as plastic particles less than 5 mm, in line with the position of the European Union. However, a number of these articles specify a lower limit for the size of MPs, choosing to also define NPs. For instance, Li et al. [45] and Song et al. [41] define NPs as particles smaller than 1 μm, while Zhu et al. [43] set the limit at 0.1 μm. The size of the MNPs significantly impacts the manner and rate of uptake by cells with smaller particles having been observed to be internalised at a greater rate through endocytic or passive uptake processes, whereas larger particles are more dependent on phagocytosis mechanisms [56, 57]. Similarly, the pathophysiological effects of MNPs have been observed to be size-dependent, potentially due to a higher surface area to volume ratio [58]. Polystyrene nanoparticles (20–50 nm), for example, induce apoptosis and inflammation in HK-2 cells [43]. Additionally, HEK 293 cells exposed to polystyrene NPs (20, 60, 100, 500, 1000 nm) showed size- and concentration-dependent toxicity, with smaller particles causing more significant viability reduction and oxidative damage [45]. Moreover, a study on mice demonstrated that microplastics (5 μm and 20 μm) accumulated in the kidney, liver and gut, with smaller particles showing higher tissue accumulation and associated toxic effects, including oxidative stress and inflammation [59]. This size-dependent toxicity highlights the need for standardised definitions to ensure consistent communication of research findings. In addition, further categorisation may be required to take into account other variables such as morphology (e.g. shape, surface characteristics and polymer matrix), additives including pigments, and particle charge. As research continues to be undertaken, it is essential for clear universal definitions surrounding MNPs to ensure they are useful for academic and clinical science while facilitating accuracy through consistency within this rapidly evolving field.

### Cytotoxicity of MNPs

**Fig. 6:**
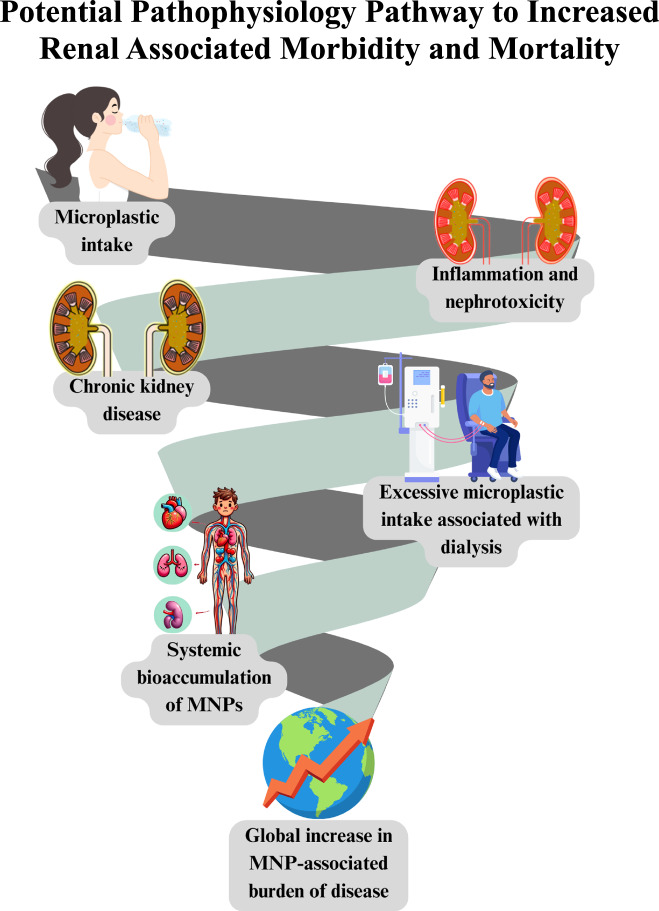
Global burden of disease. Potential mechanism by which MNPs contribute to chronic disease burden globally.

The cytotoxic effect of MNPs, and the various mechanisms by which MNPs induce cellular damage, has received increasing attention within research in recent years amongst a variety of organ systems, such as the gastrointestinal tract and cell lines, including peripheral blood lymphocytes [60–64]. To date, a limited number of polymer types and shapes have been utilised within cells lines relating to the urinary tract to investigate their potential deleterious effects. Currently, pristine polystyrene spheres are the most commonly utilised MNP model. Previously, rat models have demonstrated the nephrotoxicity of polystyrene NPs, highlighting their potential ability to induce oxidative damage and endoplasmic reticulum stress [17, 65]. The study by Li et al. [45] highlights the significant cytotoxicity of polystyrene NPs on HEK 293T cells, which found a 74.8% and 53.0% reduction in cell viability after 12 h when exposed to 60 nm and 20 nm particles, respectively. Importantly, these results may significantly underestimate the true toxic effect of MNPs. Dailianis et al. demonstrated that polystyrene NPs following degradation via ultraviolet radiation exposure, a more realistic simulation of human environmental exposure, had a significant increase in cytotoxicity [66]. Regardless, these findings are particularly concerning when compared to the cytotoxic effects of the chemotherapy drug cisplatin, commonly used to treat bladder [67], testicular [68], lung [69], ovarian [70], head and neck [71], and cervical cancer [67]. On the same HEK 293T cell line, cisplatin reduced cell viability by ~80% over 24 h [72]. The comparison underscores the potent cytotoxicity of NPs, which rivals that of pharmaceutical agents specifically designed to kill cancer cells. Increased nephrotoxicity, associated with lifelong accumulation of MNPs, has potential aetiological implications for chronic kidney disease. Of particular concern are patients who progress to requiring dialysis, which may inadvertently introduce massive quantities of MNPs directly into the bloodstream (Fig. 6) [73].

### MNP associated renal disease

Recent animal studies have elucidated multiple pathways through which these plastic particles can induce renal injury. Renal damage from disruption of the gut-kidney axis may occur as a result of increased intestinal inflammation-associated permeability following MNP ingestion. This could have the flow-on effect of leading to the translocation of lipopolysaccharides into the bloodstream and activation of the C5a/C5aR pathway [74].

Recent findings from Xu et al. further elucidate the impact of polystyrene MPs, particularly when combined with a high-fat diet, using mouse models [75]. Utilising single-cell RNA sequencing, they revealed that polystyrene MPs alongside a high-fat diet exacerbate kidney injury in mice, inducing a profibrotic microenvironment and reshaping kidney cellular components [75]. This treatment inhibited renal development and induced ROS-driven carcinogenesis, through the activation of cancer-related signalling pathways like PI3K-AKT, MAPK and IL-17 in endothelial cells [75]. These findings are similar to those of Shu et al. and Xiao et al. who reported the activation of P13K-AKT and MAPK within human HK-2 cell lines [43, 46]. Additionally, PF4+ macrophages were identified as key players in promoting renal fibrosis and a pro-tumorigenic microenvironment [75]. While MNPs have not been observed within human kidney tumours to date, their presence in 13 out of 19 bladder cancers and presence within other tumours at rates higher than adjacent healthy tissue warrants further investigation.

Adding to this, Wang et al. investigated the nephrotoxicity of polystyrene MPs in juvenile rats [17]. Their study demonstrated that oral exposure (1000 nm, 2.0 mg/kg/d for 28 days) significantly reduced growth rates and organ indices, and induced oxidative stress, inflammation and endoplasmic reticulum stress in kidneys. Histological analysis showed kidney lesions, disrupted serum biomarkers (blood urea nitrogen and creatinine) related to acute kidney injury [76], and elevated pro-inflammatory mediators (IL-1β, IL-6, TNF-α) [17]. Furthermore, they found that polystyrene MP exposure led to renal cell apoptosis and endoplasmic reticulum stress, confirmed by increased expression of apoptosis-related genes [17]. Treatments with N-acetyl-cysteine and Salubrinal somewhat alleviated these effects, highlighting oxidative stress and ER stress as central mechanisms in polystyrene MP-induced nephrotoxicity [17], while also demonstrating the potential for pharmacological treatments in future to combat the potential harmful effects of MNP exposure. The widespread physiological mechanisms through which MNPs have the potential to alter renal physiology, in combination with their demonstrated involvement in the aetiology of atherosclerosis, [77–80] also suggests MNPs may play a role in the aetiology of renal vascular diseases (such as renal artery stenosis and renovascular hypertension), as well as lower urinary tract symptoms relating to storage and voiding [81].

### MNP associated bladder disease and dysfunction

The urothelium lining of the bladder, originally viewed as a passive barrier, is now recognised as a primary transducer of specialised sensory properties, containing adrenergic, muscarinic, and purinergic receptors, and facilitating secretion of mediators including acetylcholine (ACh), nitric oxide (NO), adenosine triphosphate (ATP) and prostaglandins [82]. These mediators are vital for the urothelium’s role in modulating detrusor muscle contractions and sensing bladder fullness. The confirmed uptake of MNPs by cells, as evidenced by multiple studies [44–46, 51, 83, 84], demonstrates mechanisms by which MNPs could interfere with these critical functions.

For example, both animal and human cell line studies indicate that polystyrene MNPs cause significant mitochondrial damage, as evidenced by significantly decreased ATP production and increased oxidative stress in human umbilical vein endothelial cells (up to 82% decrease) [85], embryonic kidney (293T) and liver (LO2) cells [45]. This disruption in ATP production has implications for bladder physiology with clinical relevance for patients with urinary incontinence, due to ATPs requirement in activating purinergic receptors, such as P2X1, P2Y12, and A2b, involved in controlling the contractility and relaxation of bladder smooth muscle cells [81]. Additionally, Wang et al. [86] investigated the neurotoxic effects of polystyrene MPs on brain tissue in mice, finding that exposure to these plastics resulted in decreased levels of ACh. ACh has an important role in normal bladder function as the main stimulus for contraction of the urinary bladder smooth muscle [87]. Despite having not been investigated to date, the distension of the bladder endothelium during filling is likely to increase the permeability and, consequently, uptake of MNPs in a similar manner to pulmonary cells, potentially assisting in the exacerbation of some of these effects [88]. Regardless, the MNPs could have profound effects on urinary bladder health and incontinence, underscoring the pressing need for targeted research to understand the impact of MNPs on the urinary system.

### Implications for infections affecting the urinary tract

The presence of MNPs in the urinary tract could increase the risk of UTIs including cystitis, urethritis, and pyelonephritis, by facilitating the intracellular transport of pathogenic microbes by trojan horse mechanisms, such as the formation of a protein corona, and reducing the efficacy of the innate immune system [47,89–91]. Zhang et al. [47] demonstrated the potential for MNPs to facilitate the infection of HEK293T cells by shuttling the virus into the endo-lysosomal compartment. The ability for MNPs to facilitate infection has also been demonstrated with influenza-A within A549 cell lines [91]. The rough surfaces of MNPs provide an ideal environment for bacterial adhesion and biofilm formation, potentially leading to more persistent and recurrent UTIs [92, 93]. The widespread presence of MNPs within human testicles and semen [94, 95] also highlights the potential increased transmission risk and difficulties in the treatment of sexually transmitted infections facilitated by immune suppression and trojan horse transmission [96]. As a result, MNP-associated infections impacting the urinary tract may have significant implications for worldwide antimicrobial resistance [97].

### Limitations of the papers included in this review

This scoping review highlights a lack of research into the effects of various surface characteristics and particle charges, factors which have been shown to dramatically increase the toxicity of MNPs within other organ systems. A methodological focus on the use of Raman spectroscopy to analyse the presence of microplastics in human tissue prevents an accurate understanding of the degree of MNP contamination in humans considering smaller particles are more readily endocytosed. As a result, the dosage of MNPs within cell line studies is arguably speculative to date and may significantly depend on populations and demographics. Moving forward, the use of a multistage analysis process which aims to elucidate the physical characterisation of particles followed by chemical characterisation (e.g. scanning electron microscopy coupled with Raman spectroscopy) may assist in overcoming this limitation [98]. Additionally, the use of atomic force microscopy, although cost prohibitive, may assist in determining surface charges which have been shown to affect the cytotoxicity of NPs and the degree to which they disrupt cellular metabolism [98].

### Limitations of this review

The rapid emergence of literature in this field will necessitate a re-evaluation of this review in the near future. In addition, this review is limited by the nature of articles which were included. Specifically, while the abstracts allowed for relevant data to be extracted, they provided a limited discussion of the implications of the research and the future directions, which would assist in informing and organising further research in this field.

### Future directions and authors’ recommendations

While a limited number of human tissue samples to date have been analysed, the high rates of MP contamination warrants significant concern. To fully understand the potential implications of MNPs on the urinary tract, further research is required to detect and characterise MNPs in kidney, bladder, ureter and urethral samples. In addition, complementary research methodologies such as surveys should be employed to assist in elucidating how variables such as environments (e.g. city or rural) and lifestyle factors (e.g. food or cosmetic use) affect the level of MNP contamination. The current brevity of research in combination with the high heterogeneity of MNPs suggests that further research is required to fully characterise these exogenous particles (Table 4). This lack of basic understanding of the contamination rate of organs within the human urinary tract currently hinders researchers from undertaking research on cell lines with plastic models (e.g. polymer types, shapes, additives, etc.), similar to those found within human samples. In addition, IC50 values for MNPs within cell dose-response studies would assist in developing our understanding of the toxicity of various polymer types and morphologies while allowing for a comparison between other cytotoxic compounds.

**Table 4:**
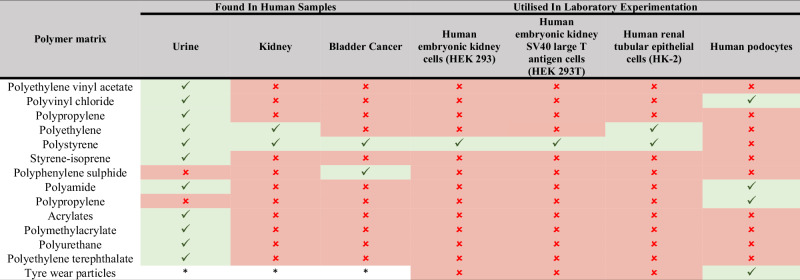
Visual display of the variety of plastics currently identified in human samples and utilised within cell line investigations.

: indicates potential gaps in the research; : indicated presence of or use of plastic polymer; *: not applicable.

## Conclusion

MNPs are increasingly being detected within human organs systems including the human urinary tract. While a limited amount of research has been conducted to date to characterise the types of plastic and their effects on the human urinary tract, growing evidence for the deleterious effects of MNPs on inflammation, cell viability, metabolism and cancer-related pathways warrants significant concern. The WHO, as well as other national and international health authorities, are urged to review current recommendations that human consumption of MPs present no threat to the health and wellbeing of the public. Moving forward, urgent research is required to (a) investigate and characterise MNP contamination, (b) understand the effects of MNPs and their impact on global burden of diseases, (c) develop solutions to prevent and treat MNP contamination within the human urinary tract, and (d) develop a standardised definition of MPs and NPs to be adopted globally, to facilitate research and assist with its translation into policy and healthcare.

## Supplementary information

Appendix 1

## Data Availability

Data is available upon reasonable request to the corresponding author.
