# Supplementary material for: Plastic induced urinary tract disease and dysfunction: a scoping review
Source: J Expo Sci Environ Epidemiol. 2024 Aug 31;35(5):770–84. doi: 10.1038/s41370-024-00709-3 (PMC12401723; doi:10.1038/s41370-024-00709-3)
Supplement: Supplementary file 1 — Appendix 1 [file 41370_2024_709_MOESM1_ESM.docx]

**Appendix 1**

**PubMed**

(Microplastic*[Title/Abstract] OR Nanoplastic*[Title/Abstract] OR Polystyrene*[Title/Abstract] OR Polyproylene[Title/Abstract] OR Polyvinyl[Title/Abstract] OR Polyethylene[Title/Abstract] OR Polyacrylonitrile[Title/Abstract] OR polymethyl[Title/Abstract] OR Polytetra[Title/Abstract] OR polyurethane[Title/Abstract] OR "Microplastics"[Mesh])

AND

(bladder*[Title/Abstract] OR kidney*[Title/Abstract] OR urine[Title/Abstract] OR Urethra*[Title/Abstract] OR "urinary tract"[Title/Abstract] OR "urinary system"[Title/Abstract] OR ureter[Title/Abstract] OR "urological"[Title/Abstract] OR "Urinary Tract"[Mesh] OR "Lower Urinary Tract Symptoms"[Mesh])

AND

(human[Title/Abstract])

NOT

(Mice[Title/Abstract] OR Mouse[Title/Abstract] OR rodent[Title/Abstract] OR Rat[Title/Abstract] OR Murine[Title/Abstract])

**Embase**

(Microplastic*:ti,ab OR Nanoplastic*:ti,ab OR Polystyrene*:ti,ab OR Polyproylene:ti,ab OR Polyvinyl:ti,ab OR Polyethylene:ti,ab OR Polyacrylonitrile:ti,ab OR polymethyl:ti,ab OR Polytetra:ti,ab OR polyurethane:ti,ab)
AND
(bladder*:ti,ab OR kidney*:ti,ab OR urine:ti,ab OR Urethra*:ti,ab OR 'urinary tract':ti,ab OR 'urinary system':ti,ab OR ureter:ti,ab OR urological:ti,ab)
AND
(human:ti,ab)
NOT
(Mice:ti,ab OR Mouse:ti,ab OR rodent:ti,ab OR Rat:ti,ab OR Murine:ti,ab)

**SCOPUS**

( TITLE-ABS ( microplastic* ) OR TITLE-ABS ( nanoplastic* ) OR TITLE-ABS ( polystyrene* ) OR TITLE-ABS ( polyproylene ) OR TITLE-ABS ( polyvinyl ) OR TITLE-ABS ( polyethylene ) OR TITLE-ABS ( polyacrylonitrile ) OR TITLE-ABS ( polymethyl ) OR TITLE-ABS ( polytetra ) OR TITLE-ABS ( polyurethane ) ) AND ( TITLE-ABS ( bladder* ) OR TITLE-ABS ( kidney* ) OR TITLE-ABS ( urine ) OR TITLE-ABS ( urethra* ) OR TITLE-ABS ( "urinary tract" ) OR TITLE-ABS ( "urinary system" ) OR TITLE-ABS ( ureter ) OR TITLE-ABS ( urological ) ) AND ( TITLE-ABS ( human ) ) AND NOT ( TITLE-ABS ( mice ) OR TITLE-ABS ( mouse ) OR TITLE-ABS ( rodent ) OR TITLE-ABS ( rat ) OR TITLE-ABS ( murine ) )

**Web of Science**

((TI=Microplastic* OR AB=Microplastic*) OR (TI=Nanoplastic* OR AB=Nanoplastic*) OR (TI=Polystyrene* OR AB=Polystyrene*) OR (TI=Polyproylene OR AB=Polyproylene) OR (TI=Polyvinyl OR AB=Polyvinyl) OR (TI=Polyethylene OR AB=Polyethylene) OR (TI=Polyacrylonitrile OR AB=Polyacrylonitrile) OR (TI=polymethyl OR AB=polymethyl) OR (TI=Polytetra OR AB=Polytetra) OR (TI=polyurethane OR AB=polyurethane))
AND
((TI=bladder* OR AB=bladder*) OR (TI=kidney* OR AB=kidney*) OR (TI=urine OR AB=urine) OR (TI=Urethra* OR AB=Urethra*) OR (TI="urinary tract" OR AB="urinary tract") OR (TI="urinary system" OR AB="urinary system") OR (TI=ureter OR AB=ureter) OR (TI=urological OR AB=urological))
AND
((TI=human OR AB=human))
NOT
((TI=Mice OR AB=Mice) OR (TI=Mouse OR AB=Mouse) OR (TI=rodent OR AB=rodent) OR (TI=Rat OR AB=Rat) OR (TI=Murine OR AB=Murine))

**CINAHL**

((TI Microplastic* OR AB Microplastic*) OR (TI Nanoplastic* OR AB Nanoplastic*) OR (TI Polystyrene* OR AB Polystyrene*) OR (TI Polyproylene OR AB Polyproylene) OR (TI Polyvinyl OR AB Polyvinyl) OR (TI Polyethylene OR AB Polyethylene) OR (TI Polyacrylonitrile OR AB Polyacrylonitrile) OR (TI polymethyl OR AB polymethyl) OR (TI Polytetra OR AB Polytetra) OR (TI polyurethane OR AB polyurethane)) AND ((TI bladder* OR AB bladder*) OR (TI kidney* OR AB kidney*) OR (TI urine OR AB urine) OR (TI Urethra* OR AB Urethra*) OR (TI "urinary tract" OR AB "urinary tract") OR (TI "urinary system" OR AB "urinary system") OR (TI ureter OR AB ureter) OR (TI urological OR AB urological)) AND ((TI human OR AB human)) NOT ((TI Mice OR AB Mice) OR (TI Mouse OR AB Mouse) OR (TI rodent OR AB rodent) OR (TI Rat OR AB Rat) OR (TI Murine OR AB Murine))
